# Antibacterial efficacy of iron oxide and silver nanoparticles against bacterial wilt pathogen *Ralstonia solanacearum*

**DOI:** 10.1038/s41598-025-19871-1

**Published:** 2025-09-23

**Authors:** Aya A. El-Lakkany, Naglaa M. Balabel, Monira M. Rageh, Mohamed S. Hanafy, Nasr Fawzy Nasr, Mohamed A. Moselhy

**Affiliations:** 1 Microbiology Department, Faculty of Agriculture, Cairo University, Giza, 12613 Egypt; 2 Potato Brown Rot Project, Ministry of Agriculture, Dokki, Giza, Egypt; 3 Bacterial Disease Research Department, Plant Pathology Research Institute, Agricultural Research Center (ARC), Giza, Egypt; 4 Biophysics Department, Faculty of Science, Cairo University, Giza, Egypt

**Keywords:** AgNPs, Antibacterial efficacy, IONPs, *Ralstonia solanacearum*, TEM, Biotechnology, Microbiology

## Abstract

The security of vegetable plants worldwide is threatened by bacterial wilts, one of the most infectious soil-borne bacterial plant diseases. This is caused by *R. Solanacearum*. Overuse of bactericides and antibiotics to combat bacterial wilt results in pesticide resistance and toxicity to beneficial living organisms. Consequently, nanoparticles are more beneficial, safe for the environment, and have strong antibacterial properties than conventional pesticides. In the present work, iron oxide nanoparticles (IONPs) and silver nanoparticles (AgNPs) were prepared by simple chemical, eco-friendly procedures, and characterized by transmission electron microscopy (TEM), X-ray diffraction (XRD), size distribution, zeta potential, ultraviolet-visible (UV-vis) absorption spectra, and Fourier transform infrared spectra (FTIR). In vitro and in vivo tests were also used to assess the nanoparticles’ antibacterial effectiveness against the phytopathogen *R. solanacearum*. The findings showed that NPs (nanoparticles) had strong antibacterial properties that changed according to concentration. The greenhouse toxicity study indicated that the NPs significantly impacted tomato bacterial wilt. The disease severity was successfully decreased by 27 and 67%, respectively, when IONPs and AgNPs were contrasted with the untreated infected plants that entirely wilted and died (100% disease severity). Therefore, as compared to infected plants, IONPs and AgNPs enhanced shoot and root length, fresh and dry weight, and chlorophyll content of tomato plants by two to five times. The findings show that the bacterial cell membranes were physically harmed by the direct attachment of NPs to their surfaces, as shown by transmission electron microscopy (TEM). In conclusion, this study provides evidence and strategies for preventing and controlling soil-borne bacterial wilt disease with an efficient and environmentally friendly metal oxide NPs. Furthermore, vegetable plant’s nutritional value is enhanced by iron, which is essential for all living things.

## Introduction

Agricultural plant pathogenic bacteria cause numerous economically significant diseases, reducing global crop yield and quality. Pathogen infection causes the buildup of metabolites and toxic poisons, posing a serious threat to worldwide food security and agricultural safety. A notable polyphagous pathogen, *Ralstonia solanacearum* causes bacterial wilting in a broad range of plant species, including tomatoes, potatoes, ground nuts, eggplants, olives, bananas, and ginger. Potatoes hold global importance as the most widely consumed staple food after maize, rice, and wheat. This highlights their critical roles in both nutrition and food security worldwide^1,2^. *Ralstonia solanacearum* detects stimuli, swims, clings to the roots, afterward gathers on the xylem vessels and obstructs the vascular system by releasing copious amounts of extracellular polysaccharides and enzymes that break down cell walls in the plant tissue. This ultimately results in host death and causes significant financial losses that amount to billions of dollars every year^3,4^. Most often, synthetic pesticides and fertilizers have been used to safeguard crops, including controlling *R. solanacearum*. On the other hand, overuse of synthetic chemicals, such pesticides and fertilizers, damages the environment, deteriorates public hygiene, and fuels the development of pesticide resistance^5^. Plant growth and development must be supported by sustainable agricultural technologies. Therefore, there is an urgent need to develop a broad-spectrum, effective antibacterial agent to control these infections.

Nanotechnology is a novel way of managing atoms and molecules at the nanoscale. Nanotechnology has developed as an interesting topic of research in modern sciences, producing many shapes of nano-size such as nanoparticles, nanorods, and nanotubes. All these nano-sized products have unique features different from their bulk components due to their exhibit high surface area compared to their small size. Numerous industries, including manufacturing, agriculture, healthcare, and cosmetics, make extensive use of metallic nanoparticles. However, even at low concentrations, they pose a serious threat to numerous pathogenic bacterial species. They impact a variety of microbial metabolic processes by acting on bacterial cells on several levels. They could rupture the membrane and cell wall, which increases cell permeability and makes it easier for antibiotics to enter the bacterial cell and change its metabolic processes. Furthermore, reactive oxygen species produced by these interactions with microbial DNA can harm the cells^6–8^.

Iron is a cofactor for many enzymes and a key element in electron chains. It is a necessary component of nearly every living organism. For photosynthesis and the synthesis of chlorophyll, plants need iron. Few bacteria can substitute other metals for iron. The amount of iron in soil affects crop productivity, plant species distribution in ecosystems, and nutritional quality. Insufficient consumption of iron led to poor health, interveinal greensickness, and limited plant development. To prevent iron, lack-induced anemia, one of the most established nutritional disorders in the world, adequate iron levels in food crops are essential. However, cells are poisoned by too much iron. The frequently limited supply of soil iron must be overcome by plants using mechanisms that increase their mobility and limit their uptake when it is present in excess^9^. Numerous studies indicate that optimal concentrations of AgNPs are essential for promoting plant growth and seed germination, boosting chlorophyll and photosynthetic efficiency, and enhancing the efficiency of fertilizer and water application. The impact of nanoparticles on plants depends on their size, composition, and amount. Additionally, plant species, growth conditions, soil characteristics, and the bioavailability of AgNPs in the soil all affect the activity of silver nanoparticles. Silver and iron nanoparticles exhibit distinct physicochemical properties that set them apart from other noble metal nanoparticles. Their broad-spectrum bioactivity encompasses antibacterial, antifungal, antiviral, anticancer, and anti-inflammatory effects. Notably, their potent antibacterial mechanisms render them a promising approach for mitigating bacterial resistance, offering a viable alternative to conventional antimicrobial strategies^10–14^.

Thus, the current study aimed to synthesize and characterize Ag NPs and IONPs, and their antibacterial potential in vitro and in vivo against *R. solanacearum*.

## Materials and methods

### Materials

In these experiments, every chemical utilized was of analytical grade and didn’t need to be further purified. Ferric chloride hexa-hydrate (FeCl_3_·6H_2_O), ferrous chloride tetra-hydrate (FeCl_2_·4H_2_O), sodium citrate (HOC)(COONa) (CH_2_COONa)_2_ (2H_2_O), ammonium hydroxide (NH_4_OH, 26% of ammonia), silver nitrate (AgNO_3_, 99.8–100% pure), Casamino acid, dimethyl sulfoxide and glycerol were acquired from Sigma Aldrich, located in St. Louis, Missouri, USA. 2,3,5-Triphenyl-2 H-tetrazolium chloride (Sigma-Aldrich T8877), Crystal violet (Sigma-Aldrich C0775) Chloramphenicol (water soluble; Sigma-Aldrich C3175), Penicillin G (benzylpenicillin sodium salt; Sigma-Aldrich P8431), Polymyxin B (sulphate salt; Sigma-Aldrich P1004) and Bacitracin (Sigma-Aldrich B0125) 1250 U). Fooding (China) supplied agar. Bacto (a trademark of Difco Laboratories, Australia) provided the yeast extract, and Oxoid (England) provided the bacteriological peptone.

### Green synthesis of silver nanoparticles

According to Li et al.^15^, silver nanoparticles were synthesized using a blueberry leaf extract. In short, in accordance with international regulations and rules, fresh mature blueberry leaves were gathered from a nearby vegetal garden at Cairo University. The leaves were then carefully rinsed in tap water before being immersed and cleaned with purified water. The leaves were then dried on filter sheets at room temperature. 200 ml of distilled water was used to extract 10 g of finely chopped leaves for an hour at 80 °C. Whatman No. 1 filter paper was afterward used to filter the combination. The yellowish filtration was thereafter placed in test tubes and frozen. 20 ml of a 5 mM AgNO_3_ solution was added to a 50 ml Erlenmeyer flask, and then 0.2 ml of blueberry extract was extra to the AgNO_3_ solution. For 20 min, the liquid was whirled at 200 rpm while heated to 90 °C on a hotplate magnetic stirrer (MSH-20D, Daihan Scientifc, Wonju, South Korea). The synthesis of the AgNPs was confirmed when the color of the stirred solution changed from colorless to dark orange or light brownish, suggesting that the AgNPs were formed by the reduction of the AgNO_3_ solution by the blueberry extract. The aqueous solution of silver nanoparticles darkened in color over time.

### Iron oxide nanoparticle synthesis

The previously disclosed approach^16^ was used to synthesis iron oxide nanoparticles (IONP). Due to its ease of use and efficiency, this approach might be the most promising. In short, 40 ml of deionized water was used to dissolve FeCl_3_.6H_2_O and FeCl_2_.4H_2_O with molar ratios of 6:5 and 5 ml of ammonia solution (28% w/v%). 4.4 g of sodium citrate was added after 10 min., and the combination was heated to 90 °C for 30 min. while being constantly stirred. To get rid of any last traces of free citrate, the precipitate was cooled and then rinsed twice with acetone. During rinsing, the material was separated from the supernatant using a permanent magnet. Finally, the sample was vacuum dried without heating.

### Assessment of nanoparticles

The harmful effect of AgNPs and IONPs on biological systems is influenced by their size, shape, and other characteristics; therefore, it is essential to characterize the synthesized nanoparticles to ascertain their properties. The synthesized AgNPs and IONP were characterized using the following methods:

The size and profile of AgNPs and IONPs nanoparticles were assessed by transmission electron microscopy (JEM 1230 electron microscope. Jeol, Tokyo, Japan) (TEM). The size distribution of the gathered nanoparticles was established using the IMAGEJ program. A dynamic light scattering device (Malvern Zetasizer, nano-Zs 90, Malvern Instruments Ltd., UK) was used to assess the hydrodynamic size distribution and Zeta potential of AgNPs and IONP nanoparticles. Using X-ray diffraction (XRD model XPERT PRO-PANALYTICAL-Netherlands), the crystal structures of AgNPs and IONPs nanoparticles were determined. A UV-Vis spectrophotometer (Jenway 6405, Barloworld Scientifc, Essex, UK) was used to perform the UV-Vis spectroscopy at wavelengths between 300 and 600 nm. An FT-IR Spectrometer (FT/IR-4100 type A, JASCO, Japan) was used to measure and analyze the structures and constituents of the dried AgNPs and IONPs at a resolution of 4 cm^− 1^ in the 400–4000 cm^− 1^ range.

### Evaluation of the antimicrobial activity

First, Isolation and identification of the pathogen *R. solanacearum* is carried out in accordance with the procedure outlined by Mikhail et al., 2024^17,18^. In summary, bacteria were isolated from potato tubers collected from El Tayarya Village- EL Beheria Governorate and displaying brown rot disease symptoms. These tubers were cleaned with tapware before being superficially sterilized using 70% alcohol and flamed. 10 ml of sterile phosphate buffer (PB 0.05 M, pH 7.0) was used to soak a section that was 5–10 mm in diameter and 5 mm thick, containing most of the cortical and vascular tissue, from the stolon end for 5–10 min.100 µl of the resultant suspension was streaked on SMSA medium ( 1.0 g, casamino acid, 10.0 g, bactopeptone, 5.0 ml glycerol and 20.0 g agar were dissolved in one litter of distilled water at pH 6.9 after sterilization add filtered 2,3,5-Triphenyl-2 H-tetrazolium chloride 0.050 g, Crystal violet 0.005 g, Chloramphenicol (water soluble) 0.005 g, Penicillin G (benzylpenicillin sodium salt) 825 U, Polymyxin B (sulphate salt) 600 000 U, Bacitracin 1250 U), plates were incubated for three days at 28 °C to isolate pure cultures of *R. solanacearum*, The colonies’ morphology on SMSA were slimy, mucoid, highly fluidal, irregular, and white colonies with pink–red centers. Additionally, immunofluorescence antibody staining (IFAS), a serological method, was also used to identify isolated bacteria according to EPPO 7/21(3) and EPPO 7/97 (1)^19,20^. Second, yeast peptone glucose agar (YPGA), was prepared as follows, 2.5 g of yeast extract, 2.5 g of glucose, 5 g of peptone, and 10 g of agar were added to 1/2 liter of distilled water to create the medium. The medium was then autoclaved at 121 °C for 15 min. Following autoclaving, the flask was let cool at ambient temperature and 2.5 ml of tetrazolium was added. The cooled molten medium was then put into sterile Petri plates at a consistent depth while maintaining sterility, and it was left for 24 h at 4 °C to check for contamination. In a laminar flow, 200 µl/ plate bacterial suspension (1 × 10^8^ CFU/ ml and optical density of 0.117) was pipetted and were spread with the help of spreader to prepare the uniform bacterial lawn except one plate without bacteria was used as a negative control. Two wells with stainless steel cork borer 10 mm diameter were punched in each plate. Sterilized distilled water was placed in two wells of the agar plate as a positive control, and 100 µL of the prepared solutions of the nanoparticles IONPs (30, 15, 7.5 and 4.5 mg/ml) and Ag NPs (2.5 and 1.25 mg/ml) loaded in separate wells in the agar plate. To guarantee uniform sample diffusion into the agar, the plates were left undisturbed for an hour. The plates were incubated at 28 ˚C for 72 h. Diameter of the inhibition zones that developed around the wells were measured by millimeters^21^.

### Assessment of ionps and ag NPs in vivo effect on *Ralstonia solanacearum*

**Fig. 1:**
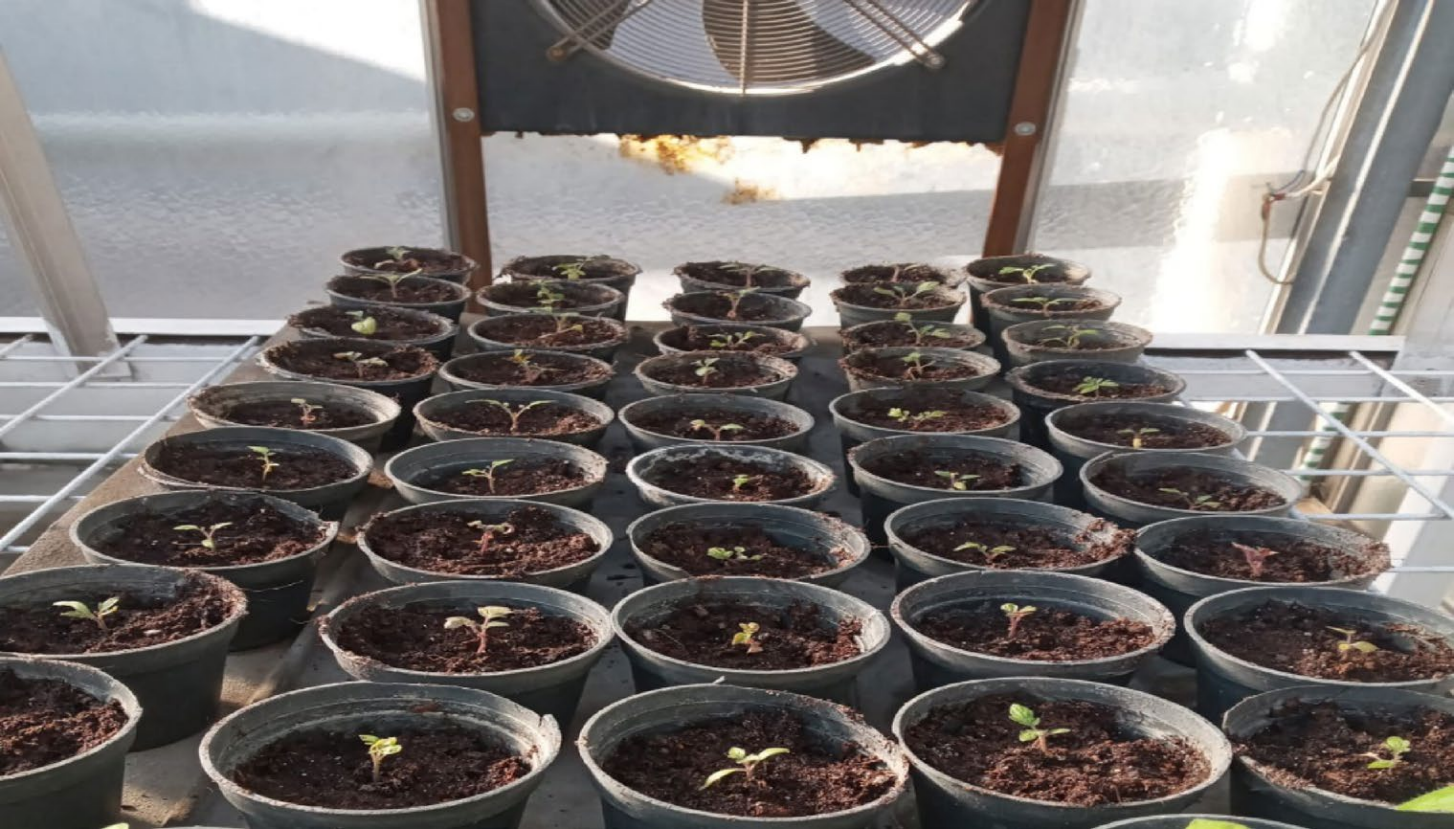
The first stage of tomato plant growth.

The experiment was conducted at the Potato Brown Rot Project controlled greenhouse Dokki, Giza, Egypt, to study the antibacterial effect of IONPs and AgNPs for the control of bacterial wilt at spring season 2024. The experiment was carried out on tomato plants (*Solanum lycopersicum* 023) as highly susceptible and rapid response to the causal agent of potato brown rot^18^. The seeds of *S. lycopersicum* were germinated in plastic crates containing peat moss for 25 days. Subsequently one seedling was transferred to each pot containing 70 g of sterilized peat moss and placed in a greenhouse (27 ± 1 ◦C, a relative humidity of 85–90%, and a 13-hour light period/11 h dark cycles) (Fig. 1). At the age of four to five-leaf (20 days), 25 ml of the *R. solanacearum* cell suspension (OD_600_ = 0.19), was used to inoculate the roots of tomato seedlings by irrigating the roots. Then, the soil in each pot was supplemented with 25 ml of water suspensions NPs at different concentration. The control pots were supplemented with sterile distilled water without NPs. Consistent tomato plants were chosen and split into ten groups (*n* = 3) as in (Table 1).

Additionally, the plants were kept and irrigated according to standard agricultural practices. After 50 days, the percentage of wilted leaves was used to calculate the disease incidence and severity using the modified Winstead and Kelman scale^22^.

$${\text{D}}{\text{.I}}=\left[ {\sum \left( {ni*vi} \right)/\left( {V*N} \right)} \right]*100$$

where N is the number of plants seen, V is the maximum disease rating (5), vi is the disease rating, and ni is the number of plants having the corresponding disease rating. A scale of 0 (no wilt symptoms), 1 (one or two wilted leaves, up to 25%), 2 (three wilted leaves, 26–50%), 3 (all leaves save the tip, 51–75%), 4 (whole plant wilted, 76–100%), and 5 (death (collapse) of complete plant) was used to determine the disease rating.

**Table 1:** Outline of greenhouse experimental groups.

Groups	Treatment
G1	Negative control (healthy plant)
G2	Positive control (infected plant)
G3 and G4 (healthy plant)	Treated with 25 ml of AgNPs at a concentration of 1.25 and 2.5 mg/ml respectively
G5 and G6 (infected plant)	Treated with 25 ml AgNPs at a concentration of 1.25 and 2.5 mg/ml respectively
G7 and G8 (healthy plant) G9 and G10 (infected plant)	Treated with 25 ml of IONPs at a concentration of 4.5 and 15 mg/ml respectively Treated with 25 ml IONPs at a concentration of 4.5 and 15 mg/ml respectively

At the end of the experiment, growth data such the plants’ fresh weight, shoot length, and root length were noted. After the plants were carefully taken out of the pots, put in designated bags, and brought to the lab, the dry weight of the plants was also determined. The plant was washed with tap water to get rid of extra moisture. The plant samples were subsequently oven-dried at 70 °C for 72 h, after which their dry weight was recorded .Additionally, fresh plant leaves were cut into 0.5 cm segments to measure the photosynthetic pigments (chlorophyll a and b), and dimethyl sulfoxide was used to extract them overnight at -10 °C. A spectrophotometer was used to test the supernatant’s absorbance at wavelengths of 648 and 664 nm. The following formula was used to determine the total amount of chlorophyll, and the amount of chlorophyll was stated as milligrams per gram of fresh weight^23^.

$${\text{Ch}}{{\text{l}}_{\text{a}}}=13.36 \times {{\text{A}}_{664}} - \,5.19 \times {{\text{A}}_{648}}$$

$${\text{Ch}}{{\text{l}}_{\text{b}}}=27.43 \times {{\text{A}}_{648}} - \,8.12 \times {{\text{A}}_{664}}$$

### Transmission electron microscope (TEM)

The process was conducted at Cairo University’s Faculty of Agriculture’s Biotechnology Laboratory in the research park (CURP). *R. solanacearum* cell suspensions (OD_600_ = 0.1) were cultivated in screw cap tubes using YPG broth medium. Treatments of bacteria were divided into three groups; 1st group treated with IONPs (30 mg/ml), 2nd group treated with AgNPs (2.5 mg/ml), while the rest group was left untreated. After that, the tubes were incubated for three days at 28 °C. Both treated and untreated bacterial cells were put on Cu (400 mesh) mesh coated with Formvar (polyvinyl formal). The film was prepared using the techniques outlined by Santiago et al.^24^, and it was analyzed using TEM and JOEL (JEM-1400 TEM).

### Statistical evaluation

One-way ANOVA and Duncan’s post hoc analysis were used to statistically analyze the collected data to compare various groups. At a P-value < 0.05, the group differences were deemed significant. The mean ± SD is used to display the data. SPSS version 26.0 was utilized to conduct these analyses.

## Results and discussion

### AgNPs and IONPs’ physiochemical characterization

**Fig. 2:**
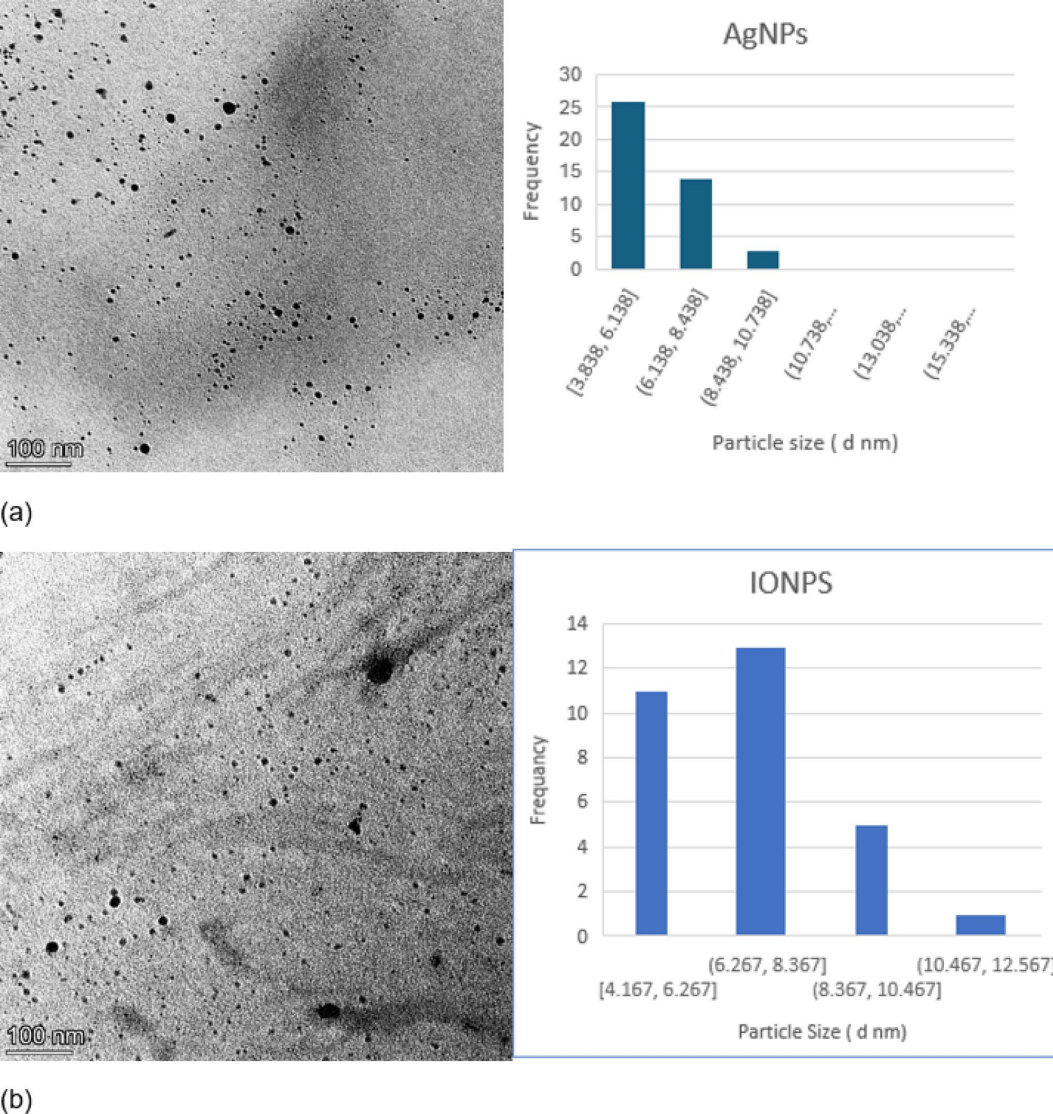
The TEM image and IMAGEJ software were used to determine the average size and size distribution of the (**a**) silver nanoparticles (AgNPs) and (b) iron oxide nanoparticles (IONPs).

**Fig. 3:**
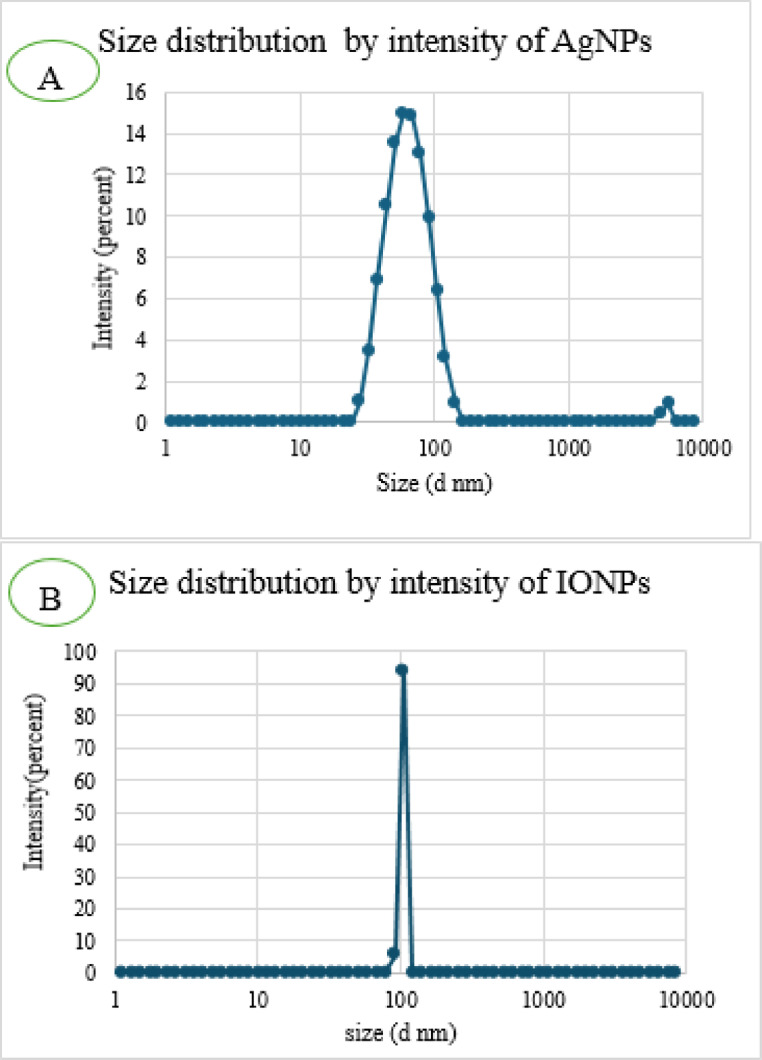
Size distribution of synthesized (**a**) silver nanoparticles AgNPs and (**b**) iron oxide nanoparticles (IONPs) by DLS.

Characterizations of AgNPs and IONP synthesized are an important marker of their stability, biodistribution, accumulation, and biofunctionalization. Nanoparticles were characterized by different techniques. The TEM images Fig. 2 demonstrate that the AgNPs and IONPs were spherical and uniformly distributed. AgNPs had sizes ranging from 4 to 15 nm, with a maximum frequency nearly 6 nm (Fig. 2a)^12^. The diameters of IONPs ranged from 4 to 13 nm, with a maximum frequency close to 7 nm (Fig. 2b)^13^. Dynamic light scattering (DLS) measurements, shown in (Fig. 3), validate this conclusion. A representative size distribution graph for AgNPs (Fig. 3a) and IONPs (Fig. 3b) is depicted in the figure. AgNP and IONP sizes are concentrated around 67 and 105 nm, respectively, with a rather narrow distribution, as the picture illustrates. In the case of DLS measurements, the surrounding water particles that are connected to the nanoparticles may be the cause of the discrepancy in the size that was observed between the two methods^25^. Additionally, the DLS recorded a polydispersity index (PDI) of 0.250 and 0.316, indicating the uniformity and good distribution of the synthesized AgNPs and IONPs in the solution, respectively. The electro-kinetic surface potential for IONPs and AgNPs was also determined using a potential analyzer, and the zeta potential values for AgNPs and IONPs were ˗22.1 ± 2.3 and ˗14.2 ± 1.2 mV, respectively. These findings showed that the nanoparticles were stable in solutions and stopped the dispersed particles from aggregating^26^.

**Fig. 4:**
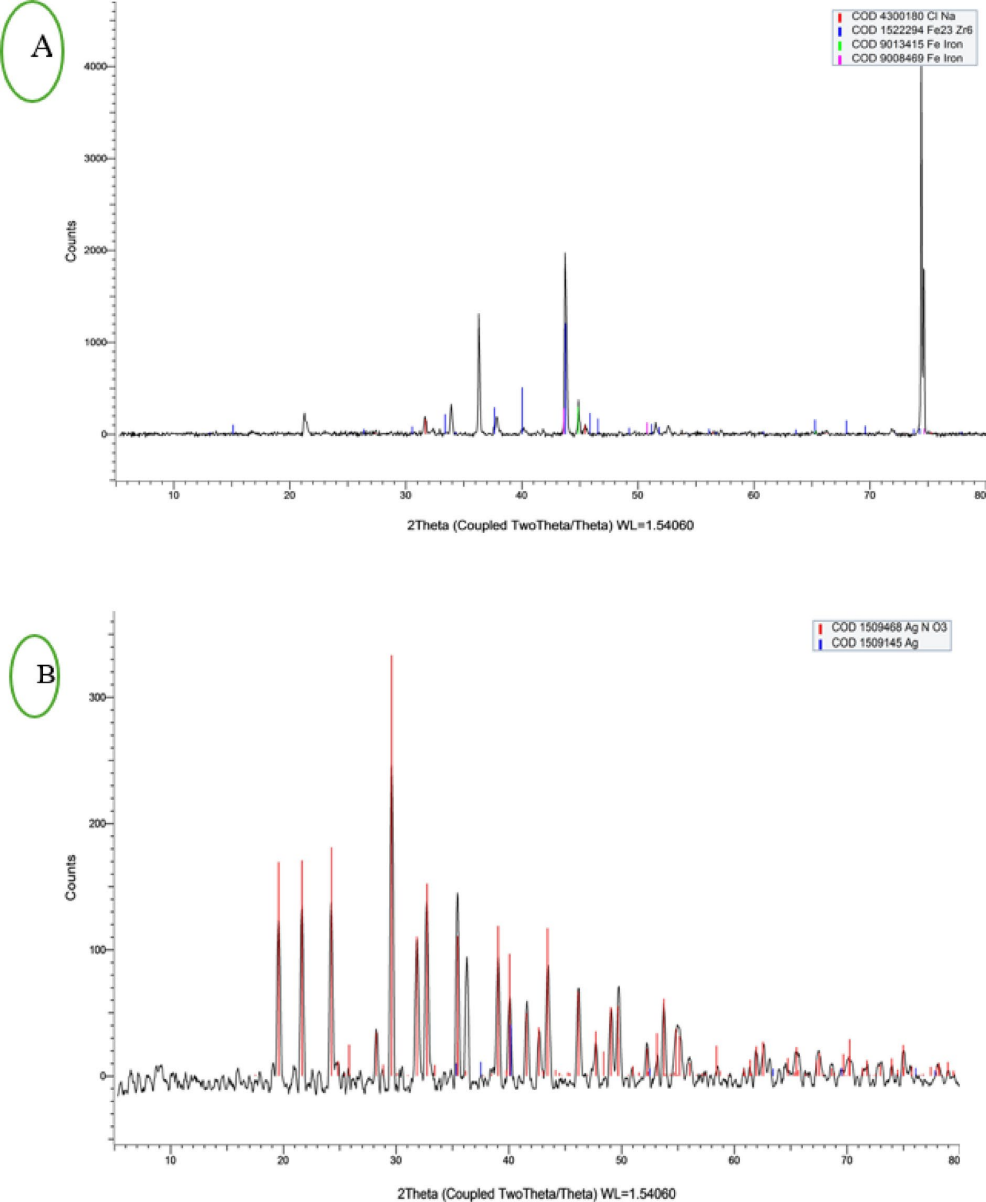
X-ray diffraction (XRD) of (**a**) iron oxide nanoparticles (IONPs) and (**b**) silver nanoparticles (AgNPs).

The XRD analysis verified the crystalline nature of the nanoparticles. Figure 4 appears the XRD pattern for IONP and AgNP nanoparticles. Reference code 9,008,469 was used to identify the cubic crystal structure of IONPs, with diffraction peaks showing at 43. 6, 50.8, 74, and 90°, indicating a pure iron nanoparticle (Fig. 4a), while reference code 1,509,145 was used to identify the hexagonal crystal structure of AgNPs, with diffraction peaks showing at 35, 37.52, 40.18, 52.49, 63.44, and 69.5°, suggesting a pure silver nanoparticle (Fig. 4b)^27–29^.

**Fig. 5:**
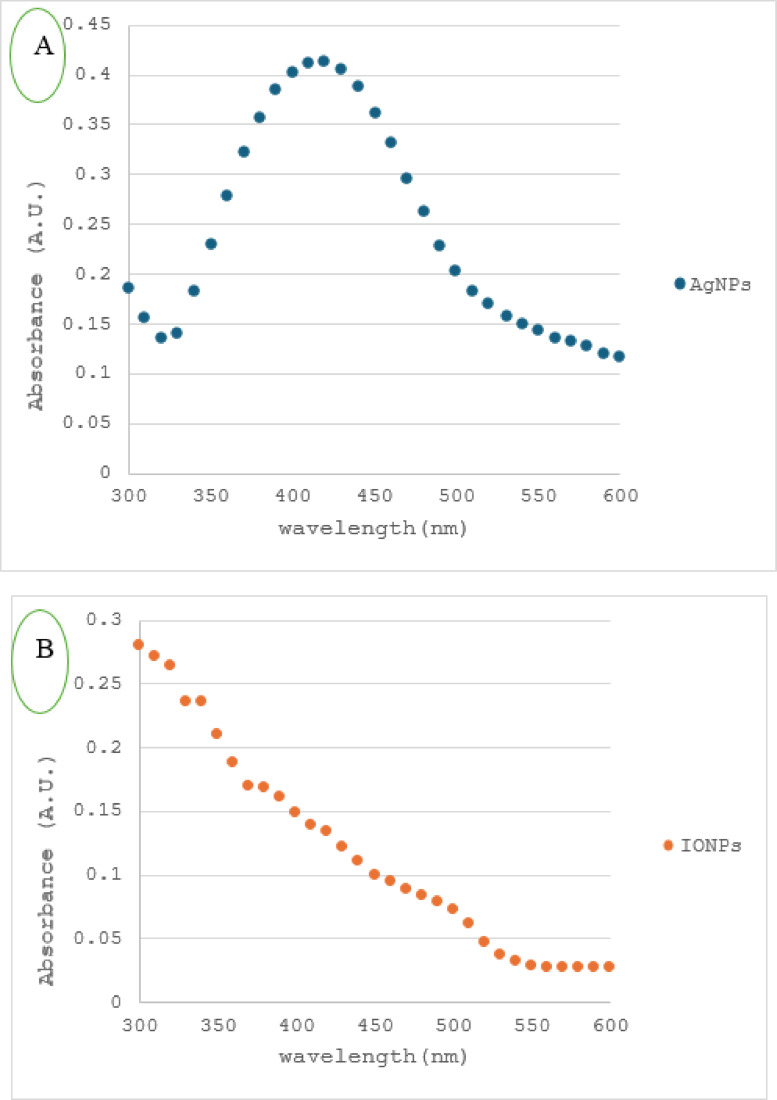
UV-visible spectra of (**a**) silver nanoparticles (AgNPs) and (**b**) iron oxide nanoparticles (IONPs).

One well-known method for describing the structure of artificial nanoparticles is UV-Vis spectroscopy. The spectra are a valuable tool for analyzing, identifying, and characterizing nanoparticles because they are sensitive to surface refractive indices, size, shape, and concentration of NPs^30^. It is therefore the easiest way to verify that nanoparticles are being produced in the ongoing study. Figure 5a depicts the formation of silver nanoparticles (AgNPs), where the band of surface plasmon resonance at 420 nm is present^31,32^, while Fig. 5b displays no observable peak for iron nanoparticles as per earlier studies^13,16,33,34^.

**Fig. 6:**
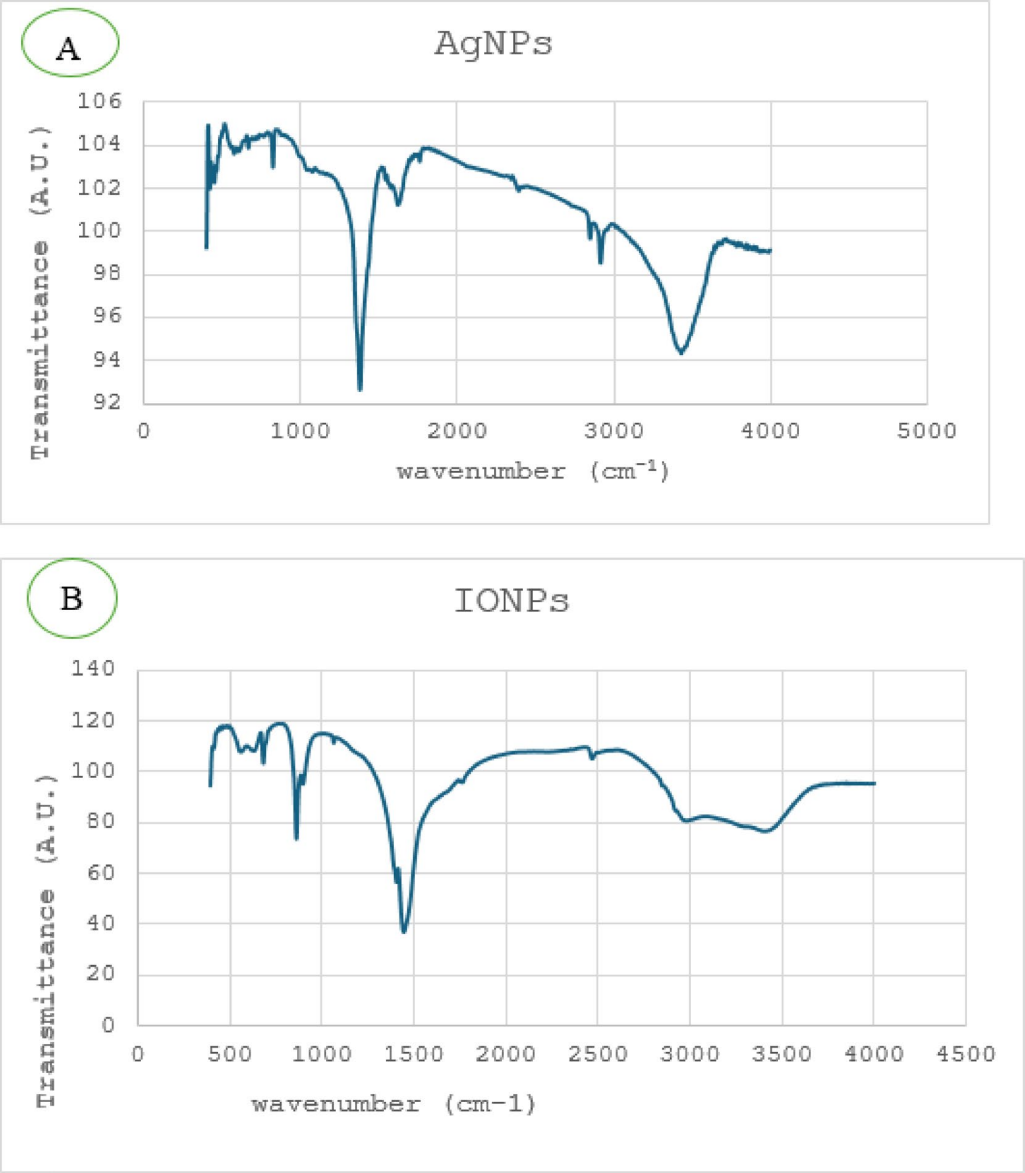
FT-IR spectra of the (**a**) silver nanoparticles (AgNPs) and (**b**) iron oxide nanoparticles (IONPs).

FT-IR has become more significant to understand the function of the efficient groups and chemical bonds present in nanoparticles. The current study used FT-IR measurements to determine which potential functional groups in the blueberry leaf extract oversee capping and effectively stabilizing the synthesized AgNPs. In Fig. 6a, the FT-IR spectra of green synthesized AgNPs illustrates that AgNPs exhibited absorption peaks at 3430, 2915, 1625, 1375, 825, and 600 cm^− 1^. The broad, solid band at 3430 cm^− 1^ was known as the O-H functional group’s strong stretching vibrations. The C-H vibrational approach was responsible for the next peak at 2915 cm^− 1^, and the stretching vibrations of cyclobenzene and alkane were responsible for the peaks at 1625 and 1375 cm^− 1^, respectively. It is possible to attribute the bands found at 820 and 600 cm^− 1^ to alkenes and aromatic rings, respectively. Similar absorption peaks were identified in the biosynthesized AgNPs’ FT-IR spectra compared to the blueberry leaf extract^15,28,35^. Figure 6b shows the INOPS nanoparticles’ FT-IR spectra. Strong stretching vibrations of the hydrogen bonds formed by OH groups were visible in the absorption band in the 3394 cm^− 1^ range. The H–C–H group’s bending causes the absorption band at 1450 cm^− 1^. Fe vibrations were identified as the cause of the absorption band at 630 cm^− 1^^36,37^.

### AgNP and IONP antibacterial activities

**Fig. 7:**
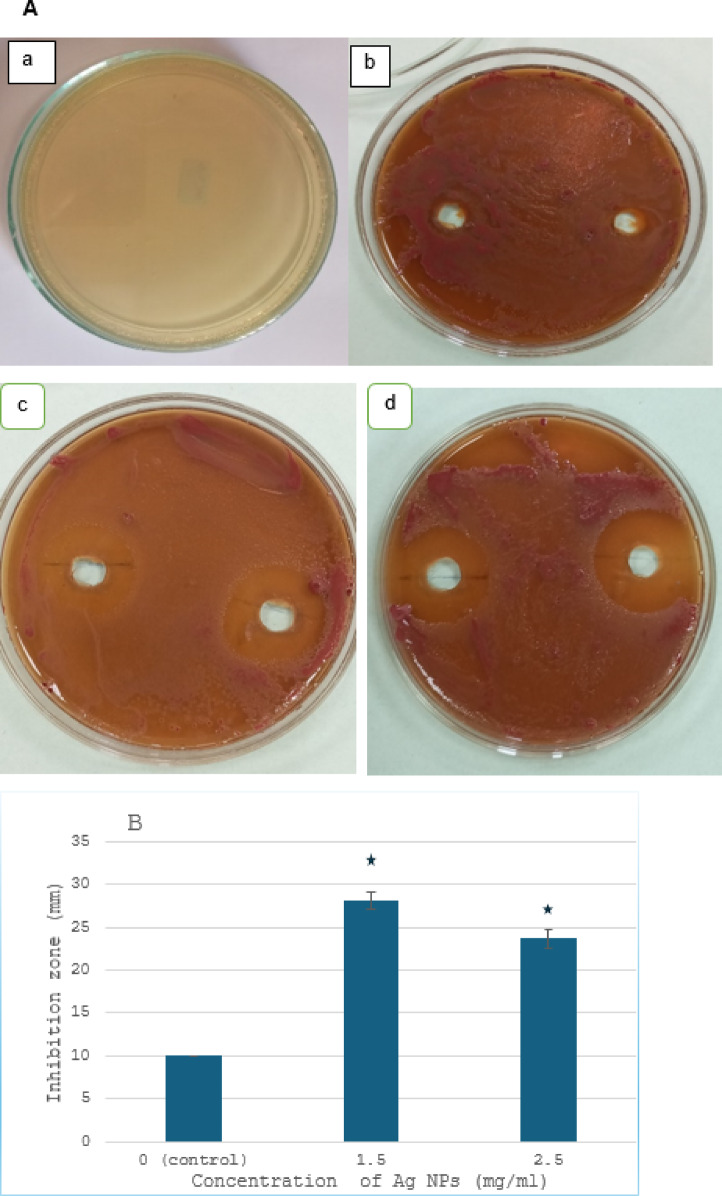
In vitro inhibitory effect by different concentration of AgNPs against *Ralstonia solanancerum*. (**A**) Diameter of bacterial growth inhibition (mm) caused by AgNPs: (**a**) negative control, (**b**) positive control, (**c**) 1.5 mg/ml, (**d**) 2.5 mg/ml; (**B**) bacterial growth inhibition zone caused by AgNPs. The data points are represented as mean ± SD (*n* = 6). Statistical difference denotes at *P ≤ 0.0001 *P* compared to positive control.

**Fig. 8:**
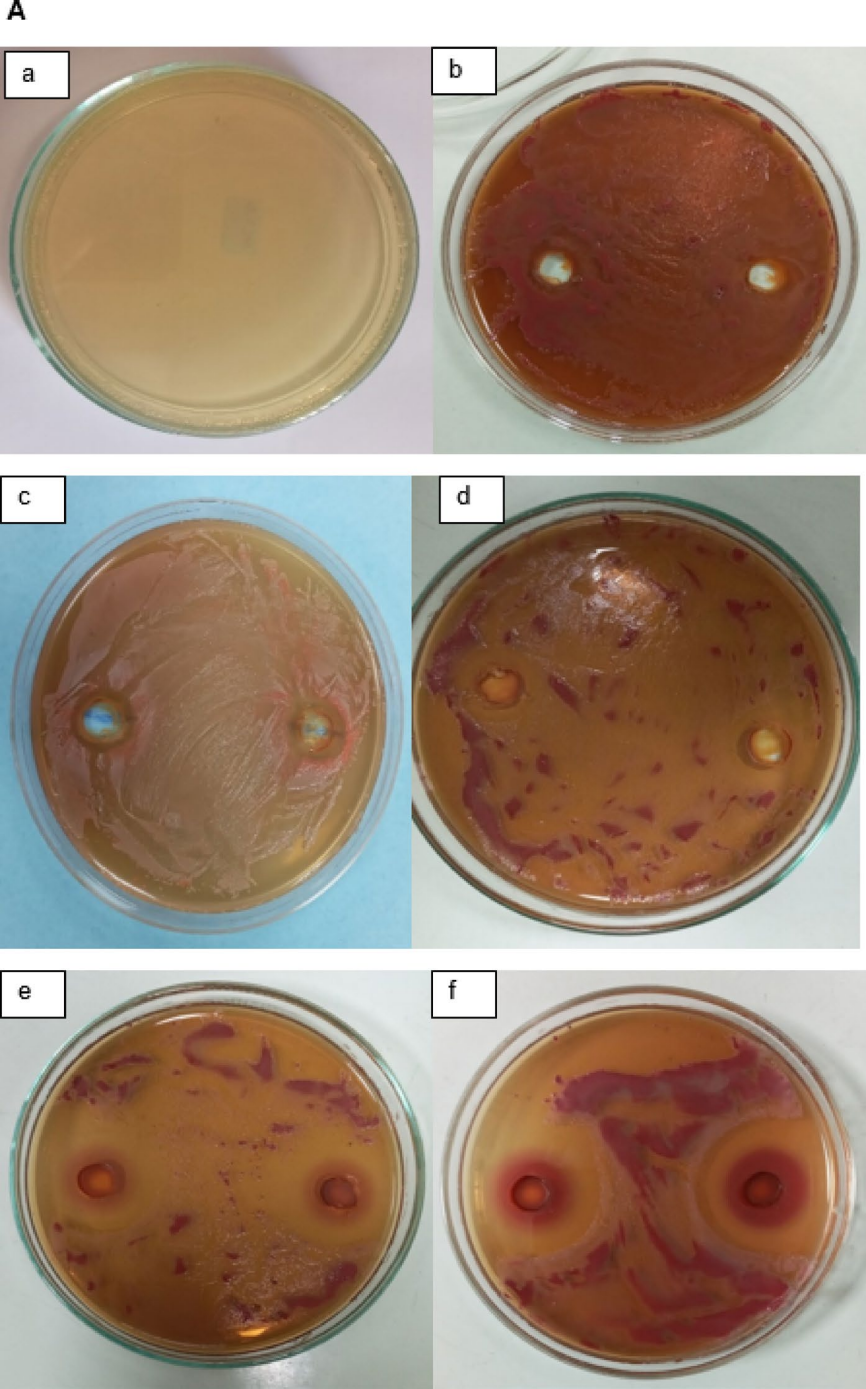

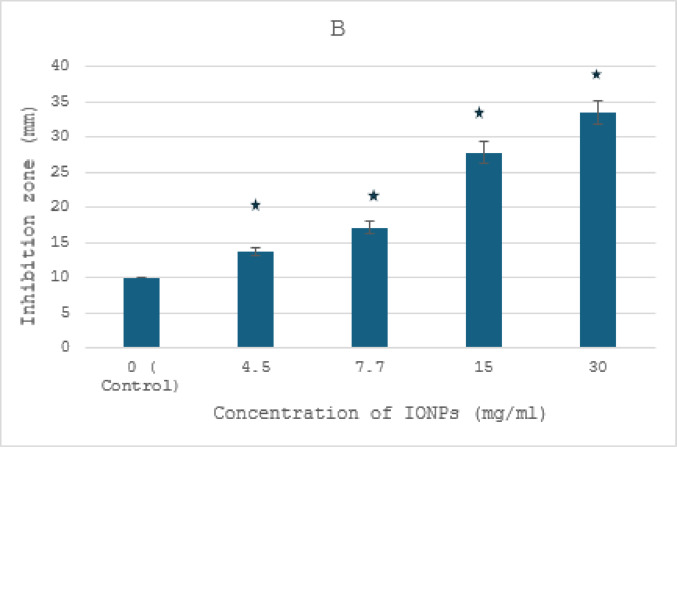
In vitro inhibitory effect by different concentration of IONPs against *Ralstonia solanancerum*. (**A**) Diameter of bacterial growth inhibition (mm) caused by IONPs: (**a**) negative control, (**b**) positive control, (**c**) 4.5 mg/ml, (**d**) 7.7 mg/ml (**e**) 15 mg/ml and (**f**) 30 mg/ml; (**B**) bacterial growth inhibition zone caused by IONPs. The data points are represented as mean ± SD (*n* = 6). Statistical difference denotes at *P ≤ 0.0001 compared to positive control.

The highly transparent areas surrounding the NP-loaded wells showed the antibacterial activity of IONPs (30, 15, 7.5, and 4.5 mg/ml) and AgNPs (2.5 and 1.25 mg/ml) against *R. solanacearum* in comparison to sterile distilled water, proving the efficacy of NPs (Figs. 7 and 8). The experiment showed that the maximum AgNP activity was 1.25 mg/ml rather than 2.5 mg/ml (Fig. 7a, b). The results showed that AgNPs did not spread throughout the bacterial medium at higher doses. However, as the concentration of nanoparticles increased, the zone of inhibition by IONPs (30,15,7.5, and 4.5 mg/ml) effectively increased, indicating that the antibacterial impact of IONPs was concentration-dependent (Fig. 8a, b). Numerous studies corroborated these conclusions by showing that the antibacterial activity of metal and metal oxide nanoparticles linked to depolarized bacterial cell membranes causes an increase in permeability, which in turn disrupts the phosphate ion and potassium ion pump, causing cellular content to leak out and ultimately leading to cell collapse. Furthermore, the NP’s antibacterial properties are linked to the bacterial cells’ elevated levels of free radicals and reactive oxygen species (ROS), which reversed redox equilibrium and resulted in oxidative stress. This illness can harm a variety of vital biomolecules and change the cells’ regular physiological functions, which could lead to cell death^28,37–40^.

### Impact of IONPs and AgNPs on tomato plant growth characteristics

**Fig. 9:**
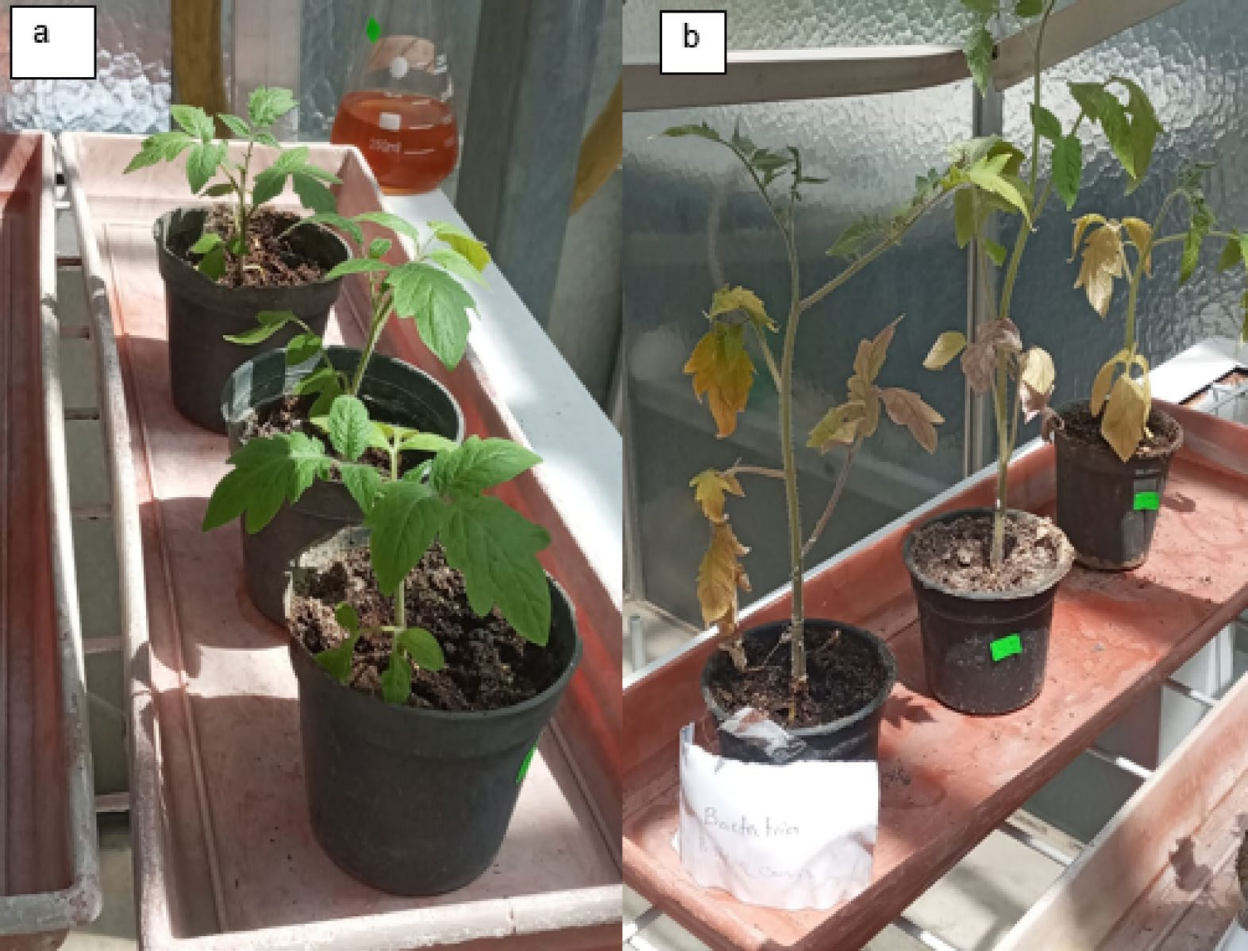
Tomato plants (**a**) healthy (negative control G1). (**b**) Infected by *Ralstonia solanacearum* (positive control G2 group).

**Fig. 10:**
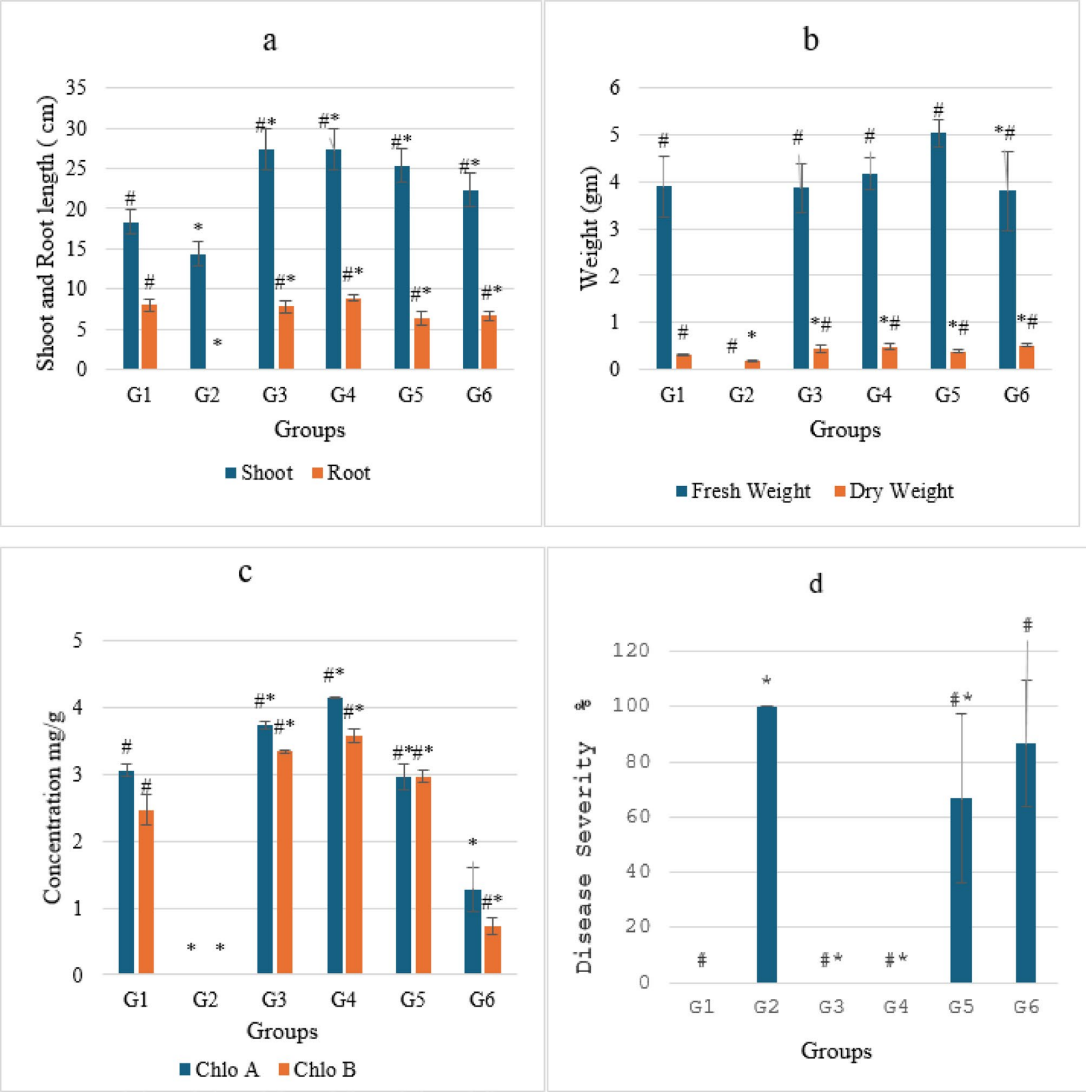
Effect of AgNPs on growth parameters of different groups from Tomato plants (G1,healthy plants, G2 infected plants, G3 and G4 healthy plants treated with 25 ml of AgNPs at a concentration 1.25 and 2. 5 mg/ml respectively and G5,G6 infected plants treated with 25 ml of AgNPs at a concentration 1.25 and 2. 5 mg/ml respectively) (**a**) shoot and root length, (**b**) fresh and dry weight, (**c**) chlorophyll a and chlorophyll b and (**d**) disease severity. The data points are represented as mean ± SD (*n* = 3). Statistical difference denotes at *P ≤ 0.05 compared to healthy plants(G1) and ^#^*P* ≤ 0.05 compared to infected plants(G2).

**Fig. 11:**
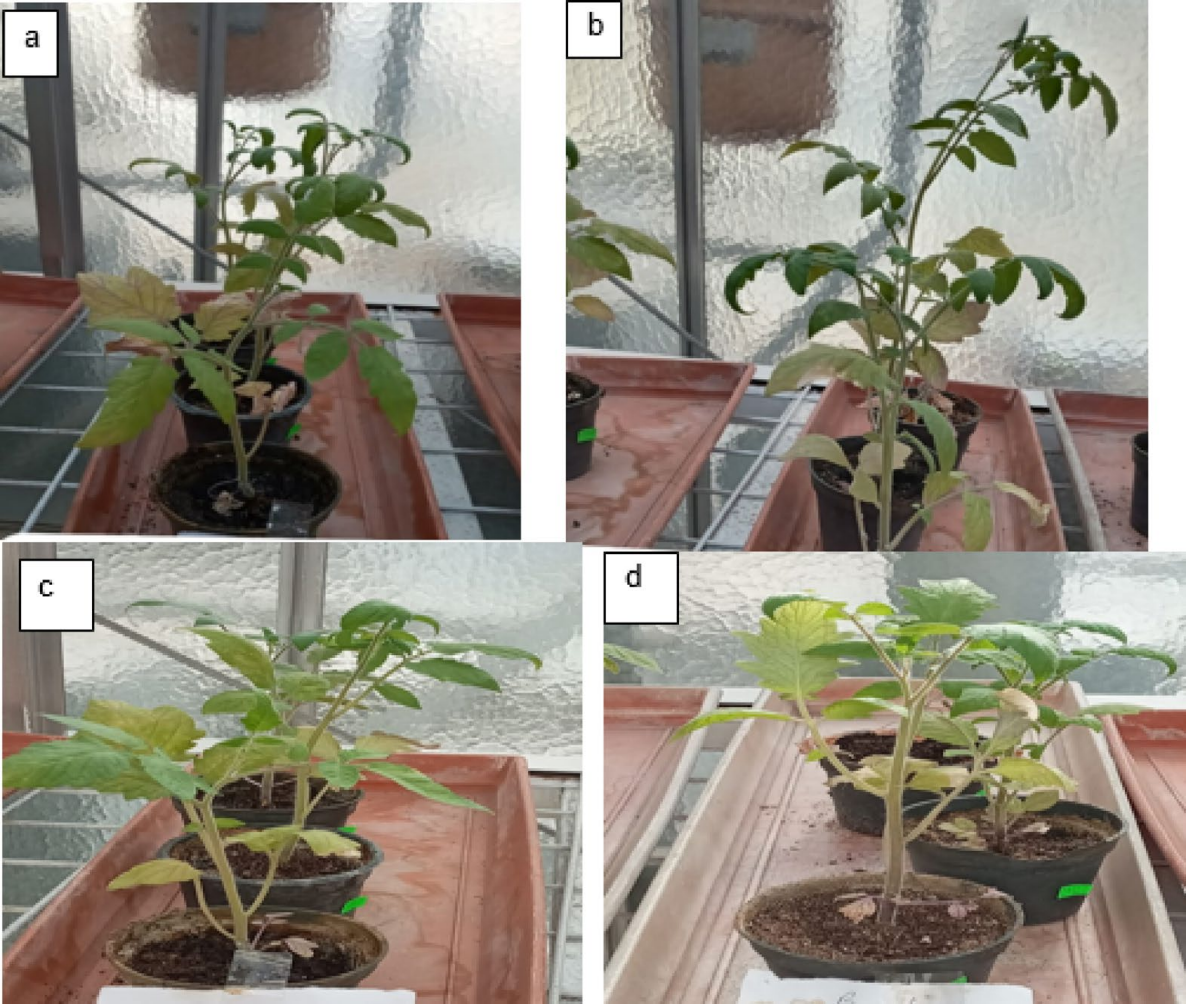
Tomato plants (**a**) and (**b**) healthy treated 25 ml of AgNPs at a concentration of 1.25 (G3 group) and 2. 5 (G4 group) mg/ml respectively. Infected tomato plants (**c**) and (**d**) treated with 25 ml of AgNPs at a concentration of 1.25 (G5 group) and 2. 5 (G6 group) mg/ml respectively.

**Fig. 12:**
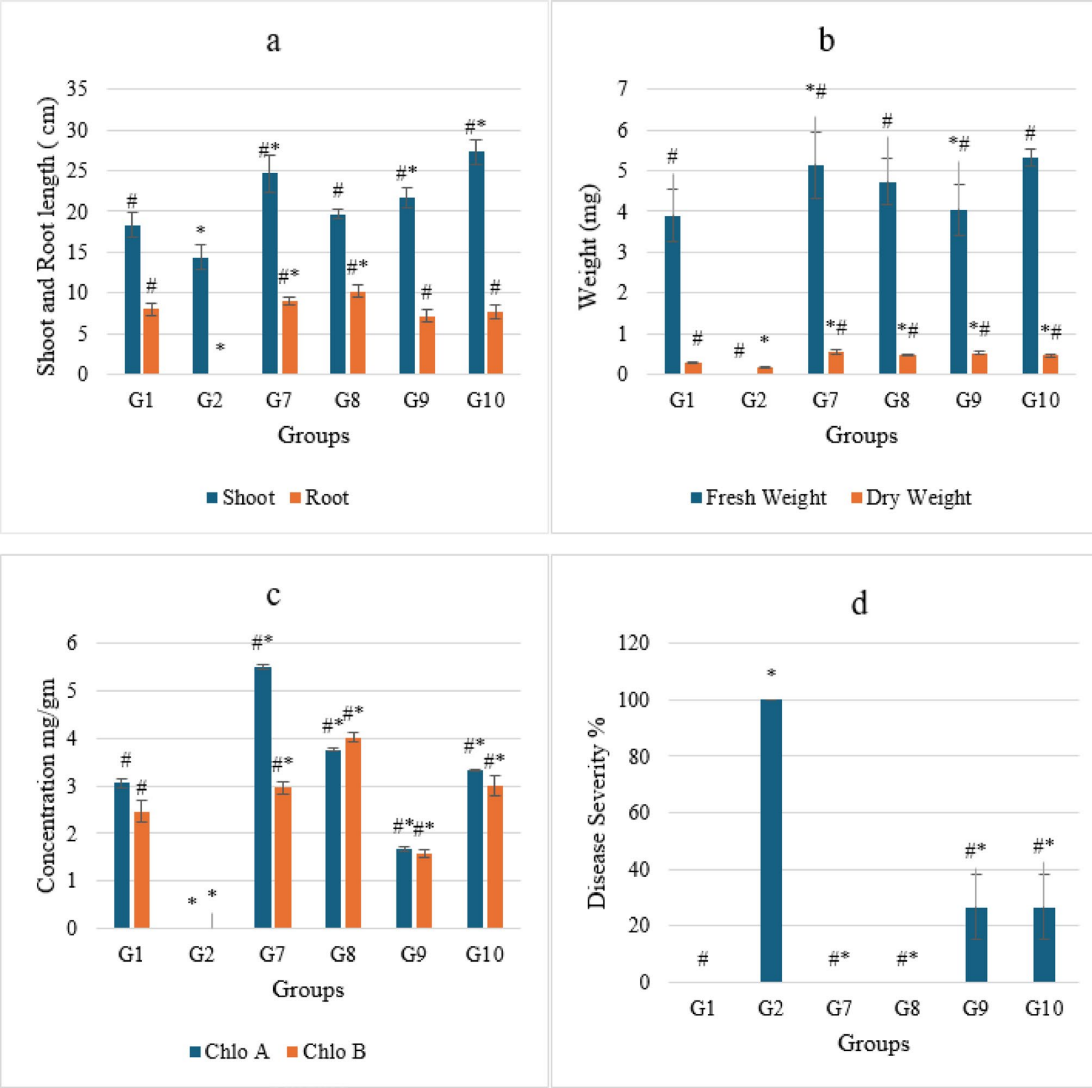
Effect of IONPs on growth parameters of different groups from Tomato plants (G1,healthy plants, G2 infected plants, G7 and G8 healthy plants treated with 25 ml of IONPs at a concentration 4.5 and 1 5 mg/ml respectively and G9,G10 infected plants treated with 25 ml of IONPs at a concentration 4.5 and 1 5 mg/ml respectively) (**a**) shoot and root length, (**b**) fresh and dry weight, (**c**) chlorophyll a and chlorophyll b and (**d**) disease severity. The data points are represented as mean ± SD (*n* = 3). Statistical difference denotes at *P ≤ 0.05 compared to healthy plants (G1) and ^#^*P* ≤ 0.05 compared to infected plants(G2).

**Fig. 13:**
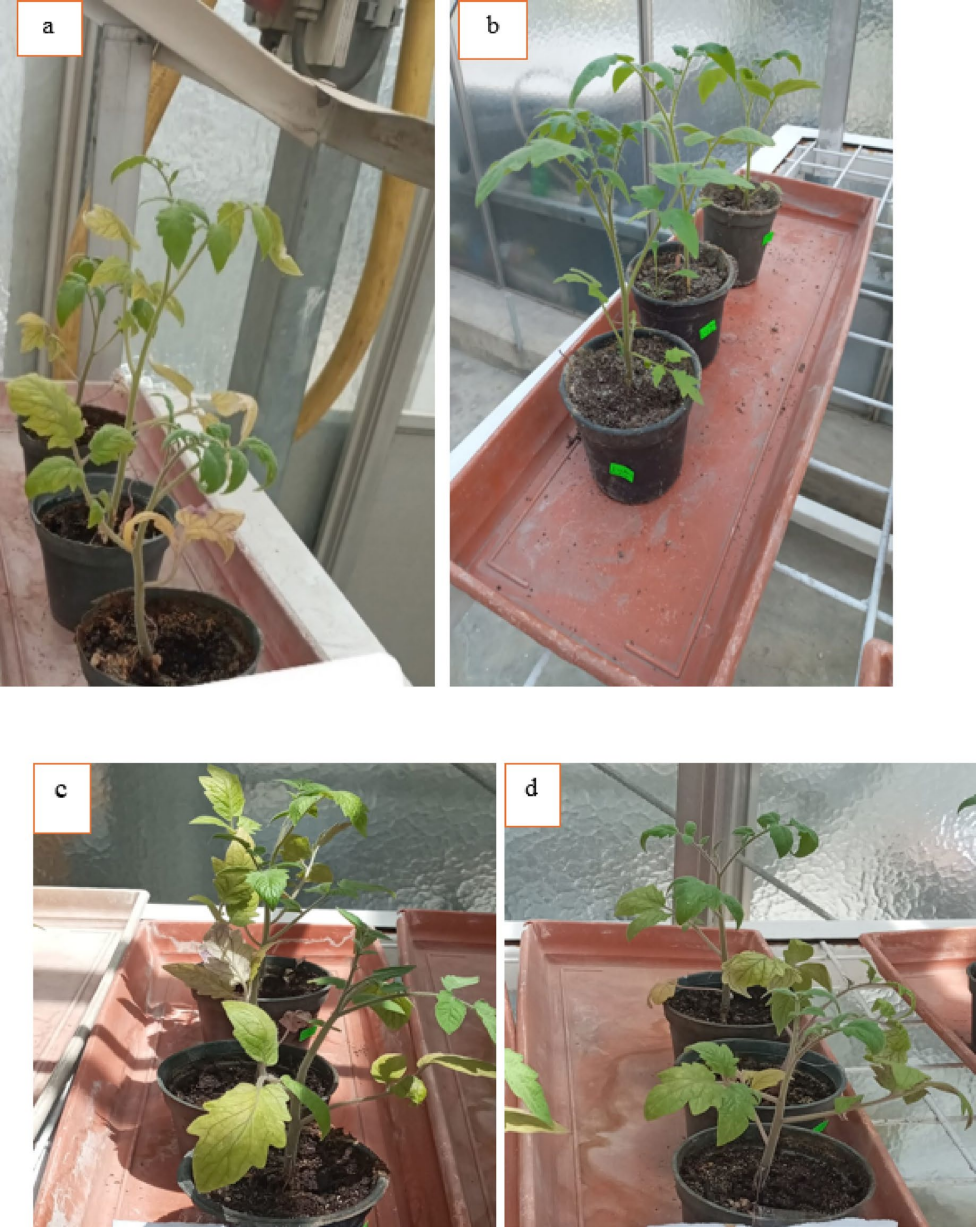
Tomato plants (**a**) and (**b**) healthy treated 25 ml of IONPs at a concentration 4.5 (G7 group) and 15 (G8 group) mg/ml respectively. Infected tomato plants (**c**) and (**d**) treated with 25 ml of IONPs at a concentration of 4.5 (G9 group) and 1 5 (G10 group) mg/ml respectively.

*Ralstonia Solanacearum* is one of the highest contagious soil-borne bacterial plant diseases, producing tomato bacterial wilt. Nanoparticles offer strong antibacterial activity, economically and ecofriendly compared to traditional insecticides. The current study used pot experiments to examine the antibacterial activity of varying IONP and AgNP concentrations against tomato bacterial wilt triggered by *R. solanacearum in vivo* in a greenhouse. Essential indicators of a plant’s general health are their morphology and photosynthetic pigments. The results demonstrated that the infected plants (G2 group) wilted and died totally (100% disease severity) after losing their fresh weight, chlorophyll content, and roots fragmented compared to healthy plants (G1 group) (Figs. 9 and 10a–d). Growth parameters (shoot and root length, fresh and dry weight, and chlorophyll a and b concentration) for both healthy and diseased tomato plants were markedly enhanced by the administration of IONPs and AgNPs. In comparison to the healthy (G1) and infected (G2) plant groups, AgNPs at concentrations of 1.25 and 2.5 mg/ml (G3, G4, G5, and G6) improved the growth parameters in tomato plants. However, the G5 group’s low concentration of AgNPs produced better results than the high concentration (G6 group), where the disease severity was 67 and 87%, respectively (Fig. 10a–d). These results were confirmed by optical photographs of the plant growth states (Fig. 11). AgNPs’ impact on growth parameters exhibited a nearly identical pattern to that shown in in vitro settings. The outcomes are entirely consistent with earlier research showing that, even at low concentrations, silver nanoparticles improve plant growth and agricultural productivity^41,42^. Furthermore, as compared to the healthy group, the IONPs groups (G7 and G8) markedly improved the growth parameters of the plants. Additionally, the treated groups (G9 and G10) displayed greater augmentation than the infected group. Furthermore, the severity of illness decreased by around 27% at 15 and 4.5 mg/ml IONPs (Fig. 12a–d). These results were corroborated by optical images of the plant growth stages (Fig. 13). These findings showed that the antibacterial activity of IONPs is concentration-dependent^43–45^.

### The mechanism of IONPs and AgNPs against *R. solanacearum*

**Fig. 14:**
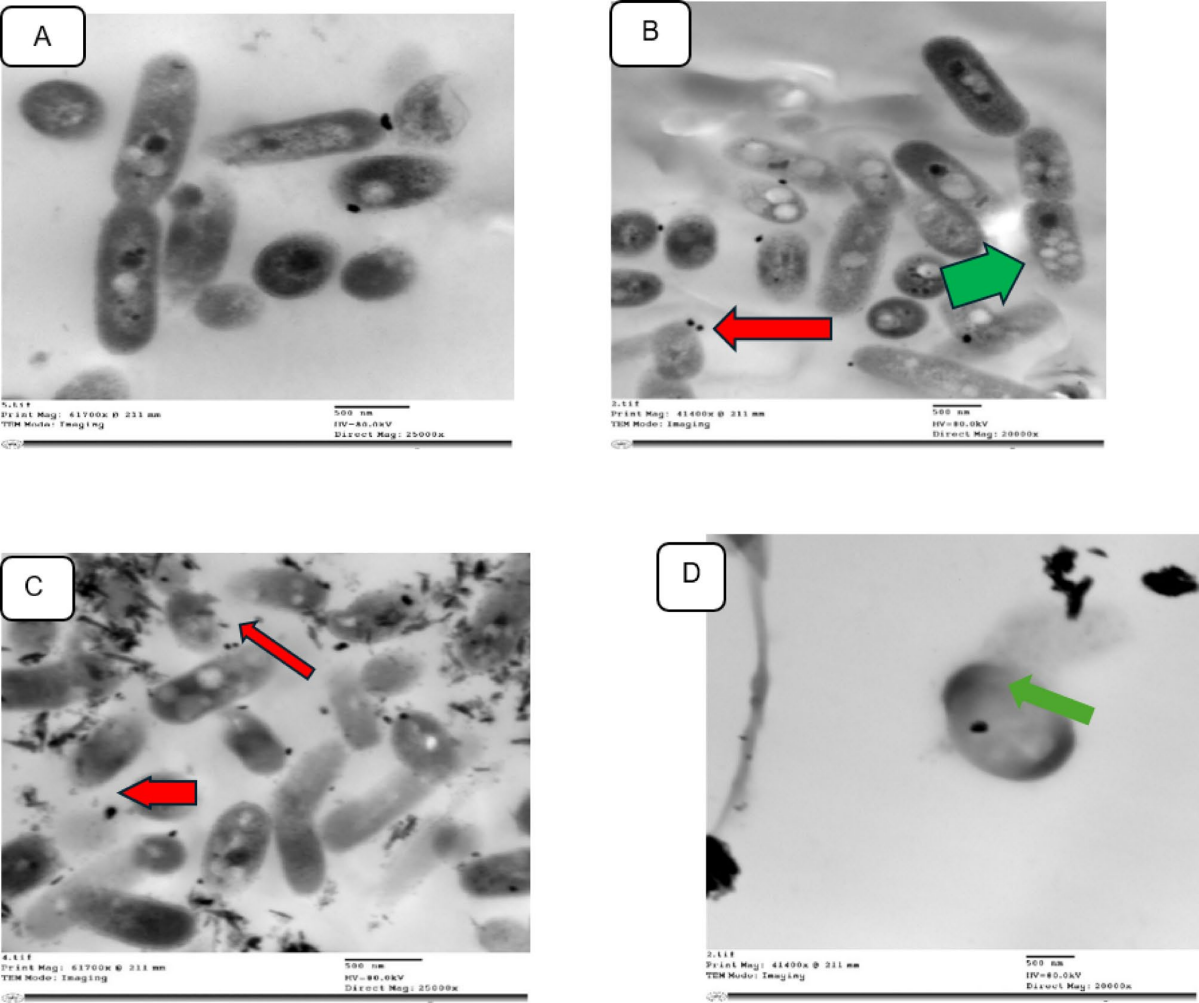
Transmission electron microscope (TEM) images of *R. solanacearum* (**a**) without treatment, (**b**) treat with 2.5 mg/ml AgNPs, (**c**) and (**d**) treat with 30 mg/ml IONPs. Scale bar = 200 nm.

The efficacy of NPs against the ultrastructural changes of *R. solanacearum* cells was investigated by atransmission electron microscope. Figure 14a shows healthy *R. solanacearum* cells with a homogeneous rod-shaped surface and an undamaged cell membrane. On the other hand, the morphology of bacteria cells treated with nanoparticles was completely different from the untreated cells. Figure 14b, c demonstrate that IONPs and Ag NPs adhered randomly and tightly to the cell surface, as indicated by the red arrow, and encouraged the production of numerous vesicles emerging from the cell wall, as indicated by the green arrow. These findings trigger broad alterations to the cell’s surface, resulting in shriveling and destruction of bacterium cell walls. These results are consistent with other studies, which stated that metal NPs have a negative impact on bacteria and shed light on the interaction between bacteria and nanoparticles. Nanoparticles’ small size allows them to cross the cellular membrane of bacteria, causing irregular gaps in the outer membrane and altering its permeability, causing osmotic imbalance and disrupting its function. When the microbial membrane loses its function, minute ions discharge first, followed by big molecules and other internal compounds, eventually leading to cell death^46–50^. Additionally, metal NPs enhanced their toxicity by the production of free radicals such as reactive oxygen species (ROS). ROS, in turn changes in intracellular oxidation-reduction reactions, metabolic stress, cellular dysfunction and lead to cell death. Furthermore, IONPs demonstrated a marked advantage over AgNPs in inhibiting *R. solanacearum* cellular component leakage, as evidenced by the green arrow in Fig. 14d. These outcomes were brought about by IONPs’ capacity to produce ROS through a variety of processes, including Fenton reactions, photocatalysis, and their magnetic properties, which produced localized heat and ultimately led to cell death. On the other hand, by covering the particle surface with phytochemicals, the green manufacturing of AgNPs lessens their toxicity^51,52^.

## Conclusion

This study evaluated the antibacterial properties of IONPs and AgNPs against *R. solanacearum* to reduce the need for pesticides. First, IONPs and AgNPs were successfully created using chemical and green processes, respectively, and characterized using various techniques. Second, the nanoparticles’ strong antibacterial activity was demonstrated by in vitro tests of their effectiveness against *R. solanacearum*. Furthermore, an in vivo investigation shows that AgNPs and IONPs decreased the severity of tomato plant disease by roughly 66 and 27%, respectively. To minimize the frequency or severity of plant diseases without adversely affecting soil fertility or non-target organisms, further research is required to identify the best nanoparticle kind and conditions.

## Data Availability

The corresponding author can provide the datasets created and/or examined during the current investigation upon reasonable request.

## Abbreviations

CURP: Cairo university research park
DLS: Dynamic light scattering
D I: Disease incidence
FTIR: Fourier transform infrared spectra
IONPs: Iron oxide nanoparticles
NPs: Nanoparticles
*R. solanacearum*: *Ralstonia solanacearum*
SMSA: Semi selective medium South Africa
AgNPs: Silver nanoparticles
TEM: Transmission electron microscopy
XRD: X-ray diffraction
YPGA: Yeast peptone glucose agar
